# Allocation Efficiency, Influencing Factors and Optimization Path of Rural Land Resources: A Case Study in Fang County of Hubei Province, China

**DOI:** 10.3390/ijerph17165898

**Published:** 2020-08-14

**Authors:** Bin Yang, Zhanqi Wang, Bo Zhang, Di Zhang

**Affiliations:** 1 School of Public Administration, China University of Geosciences (Wuhan), Wuhan 430074, China; cugyangbin2011@163.com (B.Y.); dzhang9240@163.com (D.Z.); 2 Department of Geography, University of Connecticut, Storrs, CT 06269, USA; bozhang@uconn.edu

**Keywords:** land use, allocative efficiency, influencing factors, optimal path, rural areas, Fang County

## Abstract

Land resource allocation efficiency (LRAE) is a significant indicator in weighing regional socioeconomic development. The study of LRAE can provide useful references for optimizing the layout of rural land use and conducting village planning against the background of rural revitalization strategy. Taking Fang County of Hubei Province as an example, we constructed an efficiency measurement index system based on economic, social, and ecological objectives. The slack-based measure with undesirable output (SBM-Undesirable) model and geodetector model were used to evaluate the rural LRAE, influencing factors and optimization paths from 2011 to 2017. The results suggest that: (1) the rural LRAE in Fang County shows a steady upward trend, with an average increasing rate of 9.204%. The townships in the north and south of the study area have a low LRAE value, and townships in the central area have a high LRAE value. The number of villages at low or medium-low LRAE is decreasing, and the number of villages with medium-high or high LRAE continued to increase from 2011 to 2017. (2) The spatial variation in LRAE in Fang County is affected by physical geography conditions, rural development conditions, and urban-rural relations. The impact of the proportion of primary industry and rural population has always been influential on the LRAE. Physical geography conditions have a relatively strong impact on the LRAE, but their values are decreasing. The influences of the Engel coefficient, urbanization rate and gap between the rural and urban resident’s income have been continuously enhanced. (3) All land types have obvious input redundancies, and reducing these redundancies can help achieve the optimal allocation of rural land resources. In the future, it is of significance to prioritize low-carbon and green developments, and to promote sustainable rural development.

## 1. Introduction

Land is the fundamental resource for human survival and development by providing essential goods and services, such as food production, conservation of water and soil, climate regulation, environmental cleaning [1,2,3]. However, there is a limited supply of land, therefore humans must intensely utilize land resources [4,5]. Particularly, the paradoxof limited land resources and a large population has become increasingly evident in China [6,7]. Rapid urbanization and economic development in this country have resulted in some serious issues, e.g., land resources mismatch (it refers to land misuse, such as farmland fragmentation, soil pollution, and the abandonment of cultivated land), environmental pollution, and extensive use of land resources [8,9,10]. Efficient land resource utilization is an important way to address these issues by achieving the maximum economic, social, and ecological benefits with a certain amount of inputs of resources and technology [1,11,12]. Therefore, improving land resource allocation efficiency (LRAE) is of great significance in promoting optimal utilization of land resources and guaranteeing sustainable regional development.

LRAE is a significant indicator in weighing regional socioeconomic development [13,14,15]. Scholars have conducted some research on the LRAE, including urban land allocation efficiency [13], agricultural land allocation efficiency [16,17], carbon emission efficiency [5,18], and their eco-environmental impacts [19,20,21]. As regards the research methods, location entropy [22], Cobb−Douglas production function [23], spatial Lorenz curve [24], and spatial econometric model [25] were applied to investigate the LRAE in terms of efficiency measurement, influencing factors, and their regional differences. In addition, some scholars analyzed the relationship between LRAE and socioeconomic development in regard to population growth [26,27], economic transformation [28,29], eco-environmental constraints [30,31], and resource utilization [32,33]. In general, these studies primarily focused on the LRAE of a specific land resource and have ignored the overarching efficiency of land resources. Land is a system, including numerous land resource types, e.g., construction land, arable land, woodland, water, grassland, and unutilized land. Exploring the overarching LRAE from the perspective of a land system is more objective to reveal the current situation of regional land resources and to facilitate their optimal utilization.

Recently, the concept of green and low-carbon development, which refers to a socioeconomic development mode and it places great emphasis on the importance of environmental protection, energy conservation and emissions reduction to promote sustainable regional development, continues to deepen. Some literature has focused on the studies of regional ecological security, carbon emissions, and environmental constraints and green development [30,34,35,36]. Hence, it is necessary to analyze the LRAE with the examination of eco-environmental constraints. However, few studies have discussed this topic. In this regard, we attempt to fill this gap by considering the multi-objective constraints (i.e., economy, society and ecology) of the LRAE in order to drive sustainable regional development.

Urbanization, as one of the most drastic processes of human transformation of land surface morphology, has profoundly changed regional land resource structures in China [37,38,39]. Existing literature primarily focuses on studies of the urbanization area, while the rural land, one of the important land sources for urbanization purposes, needs to be further explored. Therefore, we attempted to conduct a case study in rural areas of Fang County in Hubei Province and build a measurement index system of LRAE with regards to economic, social, and ecological objectives in this paper. Moreover, SBM-undesirable model and geodetector model were employed to investigate the rural LRAE, influencing factors, and optimization paths from 2011 to 2017. This study can provide a useful decision-making basis for optimizing rural land-use planning and rural public space governance.

## 2. Study Area

**Figure 1:**
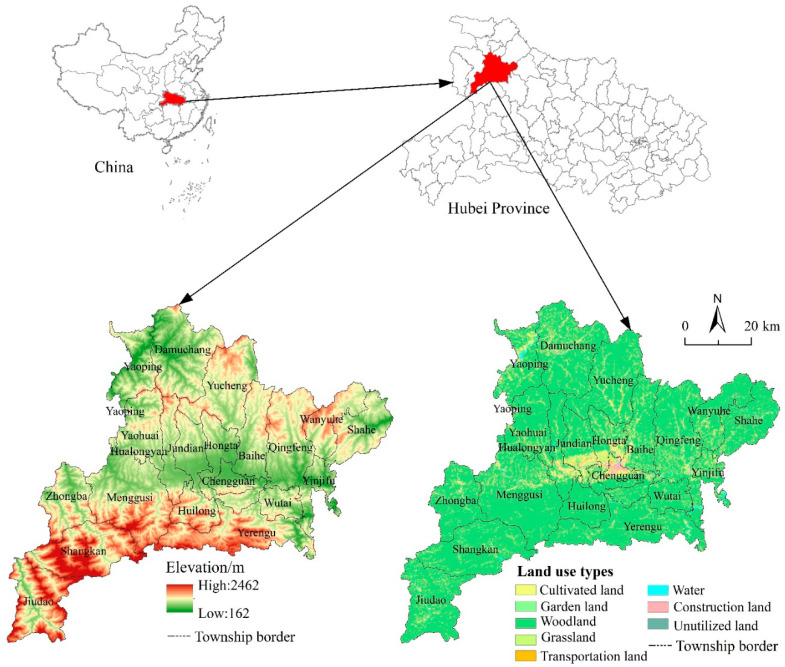
Location, elevation, and distribution of land-use types of Fang County in 2017.

Fang County is located in the northwest of Hubei Province, China. It is in the hinterland of Qinba Mountain, covering a vast territory of 5117.86 km^2^ (Figure 1). In total, there are 20 townships and 291 villages in this region. Fang County is a typical mountainous area, with woodland being the main land-use type. Other land-use types include cultivated land, garden land (In the classification of land planning, garden land belongs to a kind of agricultural land. The garden can be subdivided into orchard, tea garden and other garden), grassland, water area, construction land and unutilized land. Farming is the main agricultural type, and the main economic crops are rice, wheat, corn, soybean and cotton. In 2017, there were 106,500 households and a population of 401,200 in the rural area of the county. Food production reached 98,100 tons, and the total economic income was 8.275 billion Yuan in 2017.

## 3. Methodology and Data Sources

### 3.1. Variable Selection and Data Description

LRAE refers to the optimization degree of land-use structures under a certain technical level. The index system includes both inputs and outputs. In this paper, we selected the input variables that included three aspects: land, labor, capital. Land refers to different land resource types, including cultivated land, garden, woodland, grassland, transportation land, water areas, construction land, and unutilized land. Labor refers to the population engaged in the primary industries. Capital refers to the fixed capital investment in rural areas. The output variables include the economic, social, and ecological benefits, as well as the undesirable outputs. In this study, the total GDP in rural areas was taken as the output variable of economic benefits [40]. The social benefits were represented by the annual income of the rural residents [41]. The ecological benefits were used to measure the impact of the land resource utilization on the eco-environment, which were represented by ecosystem services values [42,43]. In recent years, low-carbon land-use transformation is an important component of “green development” [44,45]. The carbon emissions from land use in rural areas are quite different. As the main sources of carbon emissions, production and residential land have negative effects on the regional eco-environment. On the other hand, ecological land, such as woodland, grassland, and water areas, can promote environmental purification through carbon absorption, which has a positive effect on the environment [46]. Therefore, we chose carbon emissions as the undesirable output. A summary of input and output variables is shown in Table 1.

Based on results from existing research [16,35], we formulate the total carbon emission as below:

(1) $$E_{k}=\sum e_{i}=\sum A_{i}\times \delta_{i}$$

where $E_{k}$ is the total carbon emission, i is a land-use type, $e_{i}$ represents the carbon emission from land-use type i, $A_{i}$ is the area of land-use type i, and $\delta_{i}$ represents the carbon emission coefficient for land-use type i. By referring to previous studies [36,46], the carbon emission coefficients are determined as follows: 0.421 ton/(hm^2^·a) for cultivated land, −0.731 ton/(hm^2^·a) for garden land, −0.614 ton/(hm^2^·a) for woodland, −0.022 ton/(hm^2^·a) for grassland, 47.792 ton/(hm^2^·a) for transportation land, −0.253 ton/(hm^2^·a) for water areas, 33.651 ton/(hm^2^·a) for construction land, 0.032 ton/(hm^2^·a) for unutilized land.

The estimation of ecosystem services values mainly refers to the research results of Xie et al. [47]. Its formula is given as follows:

(2) $$ESV=\sum A_{k}\cdot VC_{k}$$

where ESV is the ecosystem services value (dollars), $A_{k}$ represents the area of land-use type k (hm^2^), and $VC_{k}$ is the value coefficient for land-use type k (dollars/hm^2^·a).

### 3.2. SBM-Undesirable Model

SBM-undesirable model was introduced by Tone [48]. This model was developed by the DEA (Data Envelopment Analysis) model with the non-radial and non-angle. It can measure the invalid state of the relaxation variable from both input and output dimensions, which can overcome the radial angle shortcomings of traditional data envelope analysis, making the efficiency measurement more accurate [49]. The principles are as follows. We suppose that there are n decision units in the LRAE measurement system, and each unit contains three decision vectors, including inputs, desirable outputs, and undesirable outputs. The three vectors are $x\in T^{m}$, $y^{g}\in T^{S1}$, $y^{b}\in T^{S2}$. Their corresponding matrixes are $X=\left(x_{ij}\right)\in T_{m\times n}$, $Y^{g}=\left(y_{ij}^{g}\right)\in T_{S1\times n}$, $Y^{b}=\left(y_{ij}^{b}\right)\in T_{S2\times n}$. If $X>0,\;Y^{g}>0,\;Y^{b}>0$, then we set Q:

(3) $$Q=\left\{\left(x,y^{g},y^{b}\right)|x\geq \mu X,y^{g}\geq \mu y^{g},y^{b}\geq \mu y^{b},\mu \geq 0\right\}$$

Therefore, the formula of SBM-undesirable model is:

(4) $$\rho =\min\frac{1-\frac{1}{m}\sum_{i=1}^{m}S_{i}^{-}/X_{i0}}{1+\frac{1}{S_{1}+S_{2}}\left(\sum_{r=1}^{S_{1}}S_{r}^{g}/y_{r0}^{g}+\sum_{r=1}^{S_{2}}S_{r}^{b}/y_{r0}^{b}\right)}$$

(5) $$X_{0}=\mu X+S^{-};\;y_{0}^{g}=\mu Y^{g}+S^{g};\;y_{0}^{b}=\mu Y^{b}+S^{b}$$

where ρ is rural LRAE (0≤ρ≤1); m is the number of evaluation units; $S^{-},S^{g},S^{b}$ are slack variables for input, desirable output, and undesirable output, respectively, and $S^{-}\geq 0,S^{g}\geq 0,S^{b}\geq 0$; μ is weight vector and μ≥0.

### 3.3. Geodetector Model

The geodetector model was proposed to detect the spatial differentiation of geographical elements, factor influence, and multifactor interaction recognition [50]. The model was widely used in health risk assessment, socioeconomic, and ecology and environment studies [51,52,53]. Its formula is given as follows:

(6) $$q=1-\frac{1}{n\sigma^{2}}\overset{L}{\underset{h=1}{\sum }}n_{h}\sigma_{h}{}^{2}$$

where q is the detector factors influencing the LRAE; $n_{h}$ is the number of sample units in the lower level regions; n is the number of sample units in the entire research area; L is the number of lower level regions; $\sigma^{2}$ is the variance of the rural LRAE in the entire research area; $\sigma_{h}{}^{2}$ is the variance of the lower level research area. q ranges between [0, 1]. When q=0, it means that indicator has no effect on the rural LRAE; q=1 indicates that the indicator has the strongest influence on the rural LRAE.

Previous studies on rural geography and land use have pointed out that physical geographical conditions are the basic elements that determine the spatial distribution of different types of land use, and they further impact the quantitative structure and spatial layout of land resources [11,53]. Meanwhile, rural population, socioeconomic development and industrial structures may lead to changes of land-use pattern and functions in rural areas [10,54,55]. In addition, urban-rural relations can impact the rural land-use structure to some extent [56]. Therefore, we built an indicator system to analyze the factors affecting the spatial heterogeneity of rural LRAE from three aspects, i.e., physical geographic conditions, rural development conditions and the urban-rural relationship. Specifically, physical geographic conditions refer to slope (X1) and elevation (X2). Rural development conditions refer to the proportion of primary industry (X3), rural population (X4) and Engel coefficient (X5). The urban-rural relationship refers to the urbanization rate (X6) and the gap between urban-rural per capita income (X7).

A summary of the variables representing influencing factors of rural LRAE is shown in Table 2.

### 3.4. Data Sources

Land-use data in this study was obtained from the Bureau of Natural Resources and Planning in Fang County. Based on the continuity and availability of data, we chose the duration of 2011 to 2017 as our study period. Socioeconomic data was obtained from the Statistics Yearbook of Fang County in 2012–2018. The 30-m spatial resolution DEM data was obtained from the website of the Geospatial Data Cloud, Chinese Academy of Sciences (http://www.gscloud.cn).

## 4. Results and Discussion

### 4.1. Dynamic Measurement and Analysis of Rural LRAE

#### 4.1.1. Analysis of Rural LRAE at the County Scale

**Figure 2:**
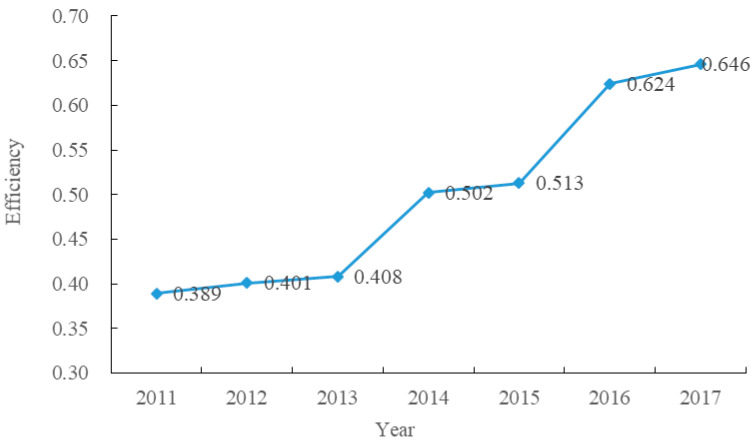
The rural LRAE at county scale of the study area from 2011 to 2017.

Based on the SBM-undesirable model, we calculated the rural LRAE at county scale of the study area from 2011 to 2017, as shown in Figure 2. The results indicated that the efficiency from 2011 to 2017 was 0.389, 0.401, 0.408, 0.502, 0.513, 0.624, and 0.646, respectively. It showed a steady upward trend, with an average increasing rate of 9.204%. It may be due to the continuous efforts on rural development by local government in recent years. Some policies, such as the new rural construction, urban-rural overall development, poverty alleviation and rural revitalization programs, optimized the land-use structures and promoted the LRAE to some extent.

#### 4.1.2. Analysis of Rural LRAE at Township Scale

**Figure 3:**
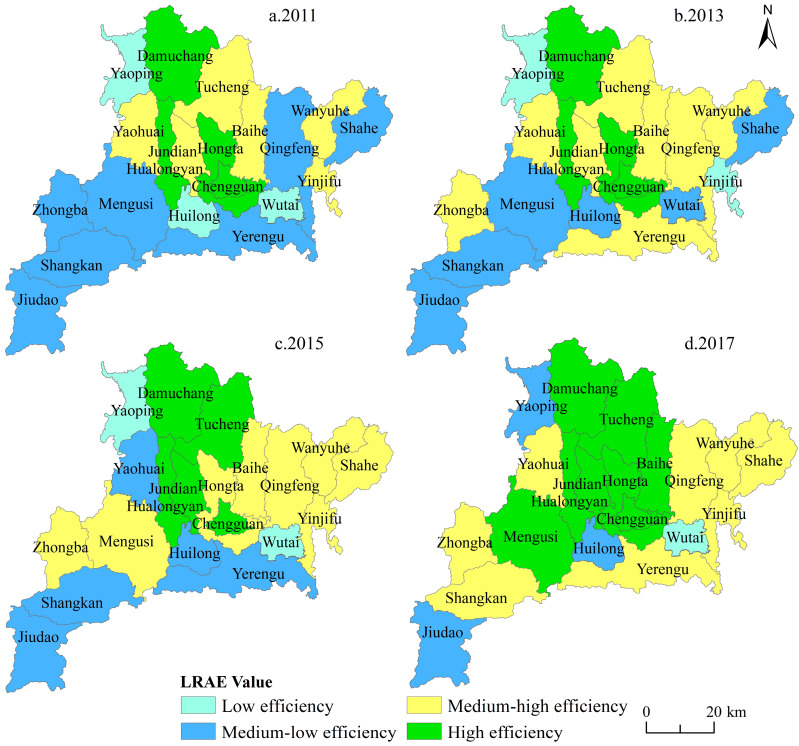
Spatiotemporal distribution of rural LRAE at township scale in Fang County from 2011 to 2017.

The rural LRAE at township scale was obtained by using the SBM-undesirable model from 2011–2017 (Figure 3). We also divided the LRAE values under the same classification scheme: low efficiency (<0.30), medium-low efficiency (0.30–0.50), medium-high efficiency (0.50–0.80), and high efficiency (>0.80). Overall, the townships in the north and south of the study area had low LRAE value, and townships in the central area had a high LRAE value. Chengguan, Hongta, Jundian, and Hualongyan in the central area always belonged to medium-high or high efficiency. In 2011, four townships had reached efficient LRAE (high level), namely, Damuchang, Chengguan, Hongta, and Hualongyan, which were concentrated in the central area of the county. Six townships had reached medium-high level, which were more evenly distributed across the county. Seven townships were at medium-low level, namely, Yerengu, Mengusi, Qingfeng, Zhongba, Jiudao, Shangkan, and Shahe. Three towns were at low level, namely, Wutai, Yaoping, and Huilong. In 2013, the high-level townships remained unchanged, but the efficiency of Chengguan, Hualongyan, and Damuchang and Hongta decreased. Eight townships were at the medium-high level, among which the efficiency of Wanyuhe, Baihe, Yaohuai, Chengtu, and Jundian increased, while Yinjifu decreased. There were six townships at the medium-low level, namely, Mengusi, Jiudao, Wutai, Shahe, Hongta, and Huilong, and their efficiencies were rising. Yinjifu and Yaoping remained the low-level efficiency. In 2015, a total of five townships were at the high-efficiency level. There were eight townships at medium-high level, which was the same as 2013. Hongta dropped from high-level to medium-high level efficiency, and Yinjifu and Shahe had improved rapidly and reached medium-high level efficiency. Five townships were at medium-low level, with the efficiency of Yerengu decreasing. However, there were two townships at low-level efficiency. The rural LRAE improved rapidly in 2017. There were eight townships reaching high-level efficiency. Hongta, Baihe, and Mengusi moved up to high level. Yerengu, Yaohuai, and Shangkan moved to medium-high level. The efficiency of Huilong and Jiudao improved, but they remain at medium-low level. Wutai was the only township with low efficiency.

#### 4.1.3. Analysis of Rural LRAE at Village Scale

**Figure 4:**
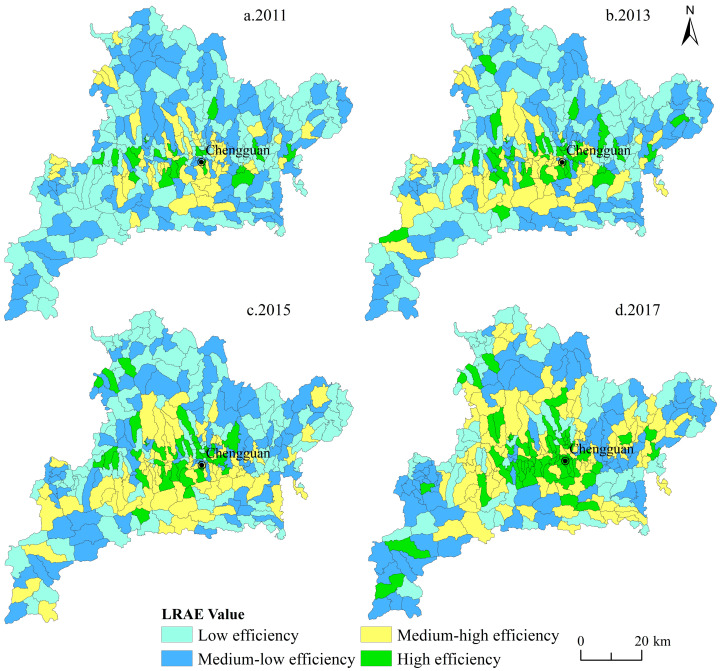
Spatiotemporal distribution of rural LRAE at village scale in Fang County from 2011 to 2017.

We took 135 villages in the study area as the research units and used the SBM-undesirable model to calculate the rural LRAE from 2011–2017. The results were analyzed based on the ArcGIS software (Figure 4). Temporally, the number of villages at low or medium-low allocation efficiency level was decreasing, and the number of medium-high or high allocation efficiency level villages continued to increase from 2011 to 2017. In 2011, 233 villages were at low or medium-low level, 204 in 2013, 197 in 2015, and only 135 in 2017. In contrast, there were 72 villages at medium-high or high level in 2011, 101 in 2013, 108 in 2015, 170 in 2017. This indicated that the LRAE in Fang County was steadily increasing during the study period. Spatially, the LRAE showed a pattern of low–high–low from north to south in the study area. Villages in Chengguan, Hongta, Jundian, and Hualongyan of the central area always belonged to medium-high or high efficiency. To the south and north of the study area are mountains with high elevation and poor transportation infrastructure. The overall economic level in these villages is low, and largely sloping land conditions have further led to unfavorable cultivation and low land productivity. These factors result in low LRAE. On the other hand, the central region is characterized by river valley plains, which are relatively flat. Fertile soils make the cultivated land rich and further improve the land-use efficiency. In addition, the administrative center of this county is the central area, which has advantages in population, economy, transportation, and locational conditions. These factors have stronger space spillover effects on the surrounding villages which drive rural development. Therefore, land resource utilization in the central area is more intensive and the LRAE is at a higher level.

### 4.2. Analysis of Influencing Factors on Rural LRAE

**Figure 5:**
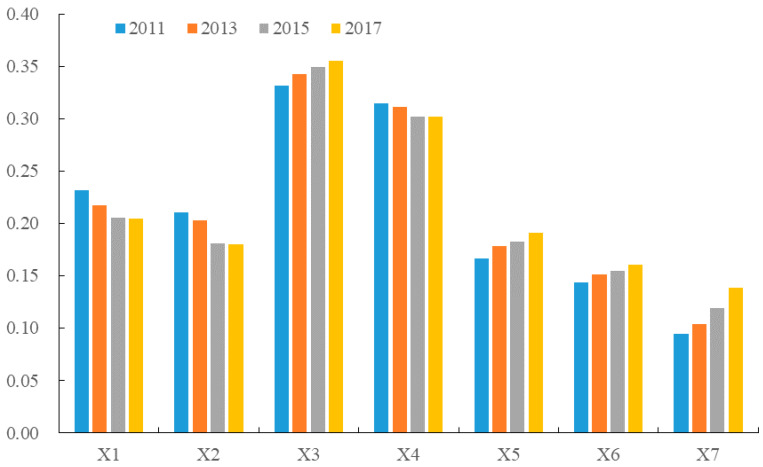
The results of influencing factors of rural LRAE in Fang County from 2011 to 2017.

We calculated the values of the factors X1–X7 based on the geodetector model (Figure 5). The results showed that the spatial variation of LRAE was affected by physical geography, rural development conditions, and urban-rural relations. The influence values of these factors varied greatly. Overall, the orders of the factors were: proportion of primary industry (X3), rural population (X4), slope (X1), elevation (X2), Engel coefficient (X5), urbanization rate (X6), and gap between urban-rural per capita income (X7). Specifically, the proportion of primary industry had the strongest influence on the LRAE. The influence value was increasing year by year. Primary industry refers to agricultural production in rural areas, which has difficulty in supporting rural development [57]. In recent years, the government has been promoting rural development in Fang County and implementing a new mode of modern agricultural production and management. Several new industries including leisure agriculture, rural tourism, and local home-staying experience have also been developing. All these measures have driven rural development and improved the LRAE to some extent.

The second important factor was rural population. Generally, population determines the demand of land for production, housing, transportation, and rural labor force. These factors further reflect the development potential of rural industry and land resource structure, which in turn affects the rural LRAE. Physical geography conditions, such as slope and elevation, have a strong influence on rural LRAE, but their importance is decreasing. The reason is that physical conditions are the basic factors determining the spatial distribution of land resources [58], which affect population distribution and land-use efficiency to some extent. However, with continuous development in rural areas, the constraints of physical geography conditions on land resource allocation are weakening. The Engel coefficient represented the economic development level of rural residents. In recent years, the living conditions have been improved significantly, which had a strong positive effect on the rural LRAE. The urbanization rate also had a great impact on the LRAE. The continuous outflow of rural residents to urban areas led to some issues, such as labor shortages, abandoned arable land, and idle rural settlements in some rural areas. These restricted the improvement of the LRAE to some extent. Additionally, urbanization took up a lot of rural land resources, and the land-use types converted from agricultural to urban land use [8]. This had a significant negative impact on the availability of rural land resources. The gap between urban-rural per capita income had the lowest impact on the LRAE, but its influence has been getting stronger. Therefore, some efforts should be made to narrow this gap and enhance rural development potential.

### 4.3. Optimal Path of Rural Land Resources Allocation

The SBM-undesirable model can not only measure the LRAE, but also improve the input-output volumes that have not reached the optimal efficiency. In this paper, we optimized the inputs and outputs of the LRAE based on the quantity and structure of rural land resources allocation of Fang County in 2017 (Table 3).

**Figure 6:**
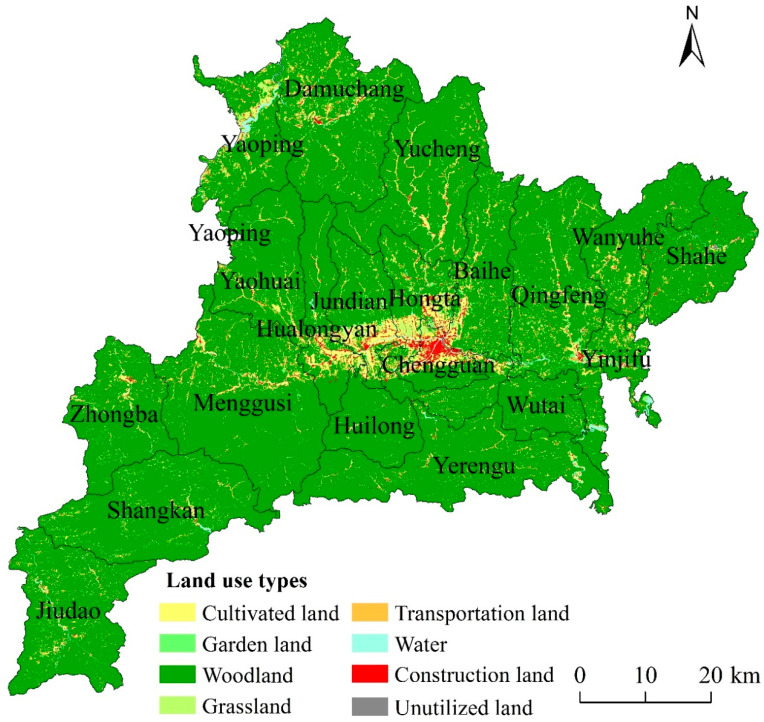
Optimal land-use map of Fang County in 2017.

Overall, there was obvious input redundancy (It means extensive utilization and waste of land resources, and reducing them can save energy, protect the eco-environment, and achieve the goal of ecological and green developments) in all land resource types in Fang County. By reducing these input redundancies, land resources can be optimized (Figure 6). Specifically, the redundancy rate of cultivated land was 8.47%, among which Baihe, Damuchang, Qingfeng, and Yerengu had the highest redundancy ratios, and Chengguan, Hualongyan, and Zhongba had the lowest ratios. With rapid urbanization in China, numerous farmers swarmed from their hometowns, causing a lot of cultivated land to be abandoned. At the same time, the requisition–compensation balance of farmland policy primarily focused on the quantity of cultivated land, but the quality and ecology were neglected, which affected the sustainable use of cultivated land [59]. Therefore, the government should pay more attention to the protection of cultivated land resources to achieve their efficient and sustainable utilization in mountainous areas. The redundancy rates of garden land, woodland, and grassland were 41.82%, 26.59%, and 35.87%, respectively. They were in a relatively high level of redundancies. The three land resource types, serving as “carbon sinks”, were important to reduce carbon emissions. In future, redundant land could be used as a reserve area for environmental protection and ecological compensation. The redundancy rates of transportation land and construction land were relatively low, with 7.74% and 11.51%, respectively. Recently, a large number of rural populations have been moving to urban areas. However, the area of transportation land and construction land has been increasing. It is necessary to manage the two land resource types and optimize their spatial layouts in the future. The degree of redundancy of the water areas was relatively high, with a rate of 24.28%. Water areas were mainly used for agricultural irrigation and drainage, which needed to fit the local conditions and be adapted with production land, e.g., cultivated land and garden land, to avoid wastage of resource allocation. Unutilized land had a redundancy rate of 27.08%, with a serious condition of redundancy. We should strengthen the exploitation and utilization of the unutilized land, and take them as important reserve sources to supplement cultivated land.

From the perspective of outputs, after optimization of the rural land resources, the total GDP of the rural areas, annual income of rural residents, and ecosystem services values were expected to increase by 13.53%, 2.29%, and 19.55%, respectively. However, the total carbon emissions would increase by 3.43%. Excessive carbon emissions have some negative impacts on the eco-environment and affect the sustainable use of land resources. Therefore, it is important to prioritize ecological and green developing (This is a socioeconomic development mode, which places great emphasis on the importance of environmental protection, energy conservation and emissions reduction to promote sustainable regional development), and to optimize the spatial pattern of land use. Only in this way can we improve the LRAE and promote sustainable rural development.

## 5. Conclusions and Policy Implications

### 5.1. Summary

In this paper, we conducted a case study in Fang County of Hubei Province and investigated the LRAE, its influencing factors and optimization path of the region from 2011–2017 by using SBM-undesirable model and geodetector model. The main conclusions are as follows:

(1) From 2011 to 2017, the rural LRAE values in Fang County were 0.389, 0.401, 0.408, 0.502, 0.513, 0.624, and 0.646, respectively. The efficiency showed a steady upward trend, with an average increasing rate of 9.204%. The townships in the north and south of the study area had low LRAE values, and townships in central area had high LRAE values. Chengguan, Hongta, Jundian, and Hualongyan in the center area always belonged to medium-high or high efficiency. Temporally, the number of villages at low or medium-low allocation efficiency level was decreasing, and the number of medium-high or high allocation efficiency level villages continued to increase from 2011 to 2017. Spatially, the LRAE showed a pattern of low-high-low from north to south in the study area.

(2) The spatial variation of LRAE in Fang County was affected by physical geography conditions, rural development conditions, and urban-rural relations. The different factors varied greatly. The impact of the proportion of primary industry and rural population has always been influential to the LRAE. Physical geography conditions, such as slope and elevation, had a relatively strong impact on the efficiency, but their values were decreasing. The influences of the Engel coefficient, urbanization rate and the gap between the rural and urban resident’s income on the LRAE have been continuously enhanced.

(3) In the process of rural land resource allocation in Fang County, all land resource types had obvious input redundancies. The redundancy rates of garden land, woodland, and grassland were at a high level, and that of cultivated land and transportation land were at a low level. Reducing these redundancies can help achieve the optimal allocation of rural land resources. From the perspective of outputs, the total GDP of rural areas, annual income of rural resident, and ecosystem services values were expected to increase by 13.53%, 2.29%, and 19.55%, respectively, after optimization of land resource allocation. However, the total carbon emissions would also increase by 3.43%. Therefore, it was of significance to prioritize low-carbon and green developments, and to promote sustainable rural development.

### 5.2. Policy Implications

Land is the fundamental resource in rural areas. It can provide several functions and services, such as rural settlements, food production, and ecological services [37,60]. It is also an important carrier for realizing rural revitalization strategy [61]. The Chinese rural land system includes the separating of the “three rights” of land, i.e., ownership rights, contractor rights, and operating rights [62,63]. Famers can have the contractor rights and operating rights, and ownership rights belong to the country. If one famer does not want to operate his or her farmland, he or she could transfer the operating rights to another famer, but the first famer also has the contractor rights. It is also called the farmland transfer in China [64]. This system can effectively avoid farmland being abandoned and improve land-use efficiency [65].

Based on the study on rural LRAE in this paper, we attempt to put forward the following policy implications to promote the utilization and management of rural land resources in China. First, the government should continue to deepen reform of the rural land system and improve the system for separating the “three rights” of rural land. We should carry out farmland transfer as soon as possible. Meanwhile, policy makers should pay attention to the cultivation of new agricultural operation entities so as to lay a good foundation for the modernized agricultural production. Second, the government should rely on the reform of the rural homestead system to revitalize idle homesteads in rural areas. Some measures, such as paid transfers or paid selling, could be taken to reduce the redundancy of rural construction land and improve the rural land market trading system to protect farmers’ legal rights. Third, comprehensive land consolidation in rural areas should be conducted to promote the overall improvement of farmland and construction land in order to revitalize inefficient land resources. In this way we could optimize land-use structure and improve land use-efficiency. Lastly, the government should promote the development of rural industrialization. We should develop new industries, such as recreational agriculture, rural tourism and cultural experiences, to further help to increase farmers’ incomes and promote rural revitalization.

However, this study has several limitations. First, the selection of the index system and the interaction mechanism between the influencing factors need to be further improved. Second, how to guide the implementation of village planning and land spatial governance based on the results of this study is unclear. Hence, future studies should focus on formulating an optimal land resource utilization plan based on spatial distribution and regional differences of LRAE, and to promote the overall development of urban and rural areas under the background of national rural revitalization.

## Figures and Tables

**Table 1:** Index system of rural land resource allocation efficiency measurement.

Criterion Layer	Indicator Layer	Indicator Description
Input variables	Land	Cultivated land (I_1_)
		Garden land (I_2_)
		Woodland (I_3_)
		Grassland (I_4_)
		Transportation land (I_5_)
		Water areas (I_6_)
		Construction land (I_7_)
		Unutilized land (I_8_)
	Labor	Population engaged in the primary industries (I_9_)
	Capital	Fixed capital investment in rural areas (I_10_)
Output variables	Economic benefits	Total GDP of rural areas (O_1_)
	Social benefits	Annual income of rural resident (O_2_)
	Ecological benefits	Ecosystem services values (O_3_)
	Undesirable output	Total carbon emissions (O_4_)

**Table 2:** The variables representing influencing factors on rural land resource allocation efficiency (LRAE).

Influencing Factors	Variables	References
Physical geographic conditions	Slope (X1)	Liu et al. (2020) [11]
	Elevation (X2)	Han et al. (2019) [53]
Rural development conditions	Proportion of primary industry (X3)	Liu et al. (2018) [10]
	Rural population (X4)	Yang et al. (2020) [54]
	Engel coefficient (X5)	Yang et al. (2019) [55]
Urban-rural relationship	Urbanization rate (X6)	Ge et al. (2020) [56]
	Gap between urban-rural per capita income (X7)	Ge et al. (2020) [56]

**Table 3:** Input and output optimization of rural land resources allocation in Fang County. Unit: %.

Township	Input (Redundancy)								Output (Optimization)			
	I_1_	I_2_	I_3_	I_4_	I_5_	I_6_	I_7_	I_8_	O_1_	O_2_	O_3_	O_4_
Baihe	15.21	31.45	31.92	27.81	8.23	20.49	8.63	28.29	14.32	2.58	18.15	3.45
Chengguan	4.60	26.90	−21.35	14.46	3.48	15.20	7.69	13.45	28.66	1.08	29.17	8.65
Damuchang	12.54	45.70	46.27	23.67	9.05	33.14	15.09	23.25	17.93	4.49	16.10	3.24
Hongta	6.34	23.26	−12.65	51.69	11.49	21.34	10.51	17.41	17.39	1.12	24.62	6.21
Hualongyan	5.54	35.42	34.87	32.97	7.42	36.01	18.72	11.42	13.40	5.76	19.47	2.36
Huilong	6.78	27.53	54.09	36.72	2.12	17.24	10.17	13.46	8.12	0.87	8.76	0.87
Jiudao	9.42	38.44	15.49	25.36	2.30	26.75	15.10	27.26	7.62	0.65	9.73	0.65
Jundian	8.46	47.52	18.92	28.96	13.70	18.76	11.23	19.77	14.07	4.31	16.95	5.25
Mengusi	1.58	58.71	14.33	26.99	9.76	22.03	15.78	20.92	18.72	1.33	35.47	4.32
Qingfeng	11.96	40.84	23.87	26.04	11.19	35.29	17.60	22.34	19.02	2.13	32.16	2.86
Shahe	8.76	35.89	34.74	43.05	16.72	39.58	20.01	29.90	14.44	2.15	23.16	3.78
Shangkan	6.75	50.12	43.20	40.71	8.41	24.53	7.69	19.14	4.66	0.36	12.45	4.91
Tucheng	9.47	34.09	23.54	38.92	16.87	22.13	8.28	18.36	9.68	1.67	15.99	4.31
Wanyuhe	8.87	43.06	26.94	43.16	4.24	32.12	9.90	17.11	11.90	2.81	23.84	1.24
Wutai	7.36	50.43	34.99	34.29	5.68	9.12	6.66	10.83	16.32	0.46	16.13	0.35
Yaoping	10.01	27.13	32.87	38.15	3.55	11.33	6.78	27.08	7.78	0.91	9.00	2.34
Yaohuai	10.07	49.72	31.28	27.41	4.20	24.87	7.79	20.81	10.84	1.56	7.28	1.85
Yerengu	12.81	49.64	35.15	54.76	4.47	23.88	8.28	20.47	20.21	3.56	28.47	3.00
Yinjifu	9.48	84.14	45.61	44.32	5.40	21.72	13.12	17.02	8.76	2.14	18.75	5.31
Zhongba	3.34	36.42	17.78	57.90	6.53	30.08	11.24	15.98	6.71	5.92	25.40	3.69
Total	8.47	41.82	26.59	35.87	7.74	24.28	11.51	19.71	13.53	2.29	19.55	3.43
